# Telemedicine Evaluation of Hip Ailments

**DOI:** 10.7759/cureus.38900

**Published:** 2023-05-11

**Authors:** Rock P Vomer, Emma York, Kristina DeMatas, Neil P Shah, Rayghan S Larick, Mantavya Punj, Raul A Rosario-Concepcion, George G. A Pujalte

**Affiliations:** 1 Family Medicine/Research, Mayo Clinic Jacksonville Campus, Jacksonville, USA; 2 Department of Family and Community Health/Department of Orthopedics, Division of Sports Medicine, Duke University, Durham, USA; 3 Family Medicine, Eastern Virginia Medical School, Norfolk, USA; 4 Family Medicine/Sports Medicine, Mayo Clinic Jacksonville Campus, Jacksonville, USA; 5 Family and Community Medicine, Mayo Clinic Jacksonville Campus, Jacksonville , USA; 6 Family and Community Medicine, Eastern Virginia Medical School, Norfolk, USA; 7 Family Medicine, Mayo Clinic Jacksonville Campus, Jacksonville, USA; 8 Physical Medicine and Rehabilitation, Mayo Clinic Jacksonville Campus, Jacksonville, USA; 9 Family Medicine/Orthopedics/Sports Medicine, Mayo Clinic Jacksonville Campus, Jacksonville, USA

**Keywords:** orthopedic exam, virtual encounter, functional exam, telehealth, hip

## Abstract

Background

During the coronavirus disease 2019 (COVID-19) pandemic, telemedicine has provided new means of patient care while still allowing for physical examination and history to be obtained. Hip ailments are a common musculoskeletal problem leading to limited function. Today, we lack a standardized telemedicine hip evaluation protocol.

Aim

The aim of this manuscript is to provide an efficient means of extracting relevant information while performing telemedicine examinations of the hip.

Methods

The authors have created a step-by-step evaluation guide for physicians to evaluate hip complaints, including inspection, palpation, range of motion, strength testing, functional assessment, gait analysis, and special testing, with images of each maneuver.

Results

We have developed a table of evaluation questions and instructions and a glossary of images of each maneuver to facilitate hip examination via telemedicine.

Conclusions

This manuscript provides a structured template for performing a telehealth examination of hip ailments.

## Introduction

With any evaluation, patient history is extremely important; telemedicine is no exception. Key elements of the history, including onset, duration, and mechanism of injury, alleviating and inciting factors, and previous surgeries, should be documented in any joint evaluation [1]. The history allows the clinician to develop a differential diagnosis that will be supported or refuted based on physical examination and diagnostic studies. Further information elicited during the physical examination is aimed at uncovering a suspected injury or condition, creating a comprehensive differential diagnosis [2]. The hip is a large weight-bearing joint, and hip pain is a common cause for patients to seek care. A tailored physical examination to evaluate a hip complaint involves inspection, palpation, range of motion (ROM) assessment, strength testing, functional assessment, and gait analysis, and special testing to develop a differential diagnosis [3]. Most information can be obtained through a video or telephone encounter with a patient [4].

The use of video and telephone consultations is becoming more common because it allows increased access for patients as there is no need to travel [5]. The use of telemedicine services as a whole has accelerated during the coronavirus disease 2019 (COVID-19) pandemic [6]. Despite this increased access, an evaluation via telemedicine poses challenges to both the patient and clinician as inherited nonverbal indicators of pain and change in function cannot be observed in the same way as they would during an in-person visit. These small clues allow an experienced clinician to differentiate between common and rare pathology, while also rapidly adjusting the scope of their differential diagnosis to account for concerns beyond the chief complaint [3]. While a history can still be elicited over the telephone, the examination has now shifted to the hands of the patient with responses that are self-reported. However, these responses may lack the details of how a joint moves or the tactile feedback involved in special testing, the responses are still highly valuable as they often focus the clinician on the patient’s perceived functional deficit [7,8]. Through creativity and teamwork with the patient, it is possible to conduct a thorough examination and provide effective care using telemedicine services.

## Materials and methods

Inspection

The examination starts by instructing the patient to stand with their feet shoulder-width apart, preferably in tighter-fitting athletic garments. Postural assessment should include anterior, lateral, and posterior views. Inspect for asymmetry in the anterior superior iliac spine or iliac crest height and rotation. Estimate the Q angle of the knee. If the angle is excessively increased or decreased, it can play a role in alerted force absorption on the lower kinetic chain and can be a source of hip pain. This assessment can be completed from the anterior and posterior views. From the lateral view, inspect for the amount of lumbar lordosis or if a sway back is present. Next, ask the patient to point to the area of maximal pain and then draw with their finger the radiation pattern. Observe for the classic C sign (patient will cup their hand around the area above the greater trochanter). Assess for erythema, swelling, or frank deformity. If the patient is with a friend or family member, have that person hold the camera to assess the patient’s gait pattern.

Palpation

**Figure 1:**
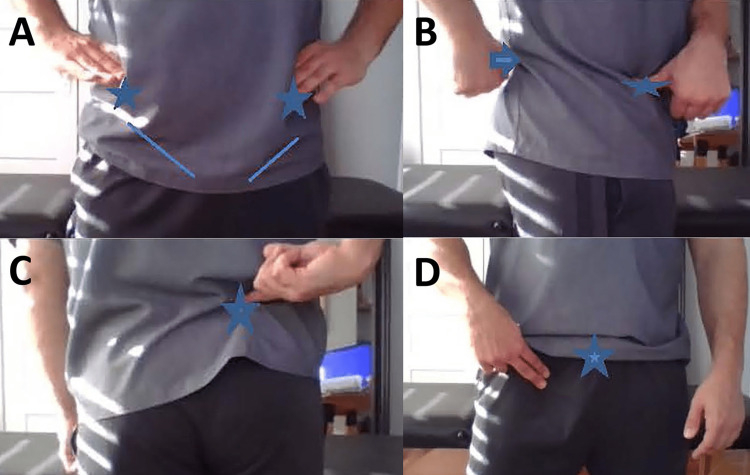
Hip evaluation by telemedicine: inspection and palpation A. Superficial anatomical landmarks of the anterior hip. The patient’s hands are placed over the iliac crests. The anterior superior iliac spine (stars) and inguinal ligament (lines) are useful landmarks to have the patient identify. A difference in hand height on the iliac crests could represent a pelvic sheer. B. Superficial anatomical landmarks of lateral hip. The anterior superior iliac spine (star) and posterior superior iliac spine (arrow) are useful lateral landmarks for assessing innominate rotation as a cause of hip pain. C. Fortin finger test. Instruct the patient to point where they experience the most pain. If it is located at the sacroiliac joint (star), this is a positive Fortin finger sign. D. Palpation of the femoral crease and pubic symphysis. A palpable mass below the inguinal ligament, as indicated by the patient’s fingers, may suggest a possible femoral hernia. Pain on palpation at the pubic symphysis and insertion of the rectus abdominis (star) could indicate possible athletic pubalgia.

Have the patient palpate the anterior superior iliac spine, posterior superior iliac spine, tensor fasciae latae, pubic symphysis, ischial tuberosity, and greater trochanter for pain (Figure 1A-1C). The patient is instructed to palpate each landmark by moving his/her hand in a clockwise manner starting with the hand on the top of the iliac crest. Have the patient palpate and comment on the subjective tone of the muscle mass, temperature, and sensation of the anterior compartment of the thigh, medial aspect of the hip, and gluteal region. Instruct the patient to palpate boney prominences on the pubic symphysis, greater and lesser trochanters, ischial tuberosity, and piriformis (Figure 1D).

ROM assessment

**Figure 2:**
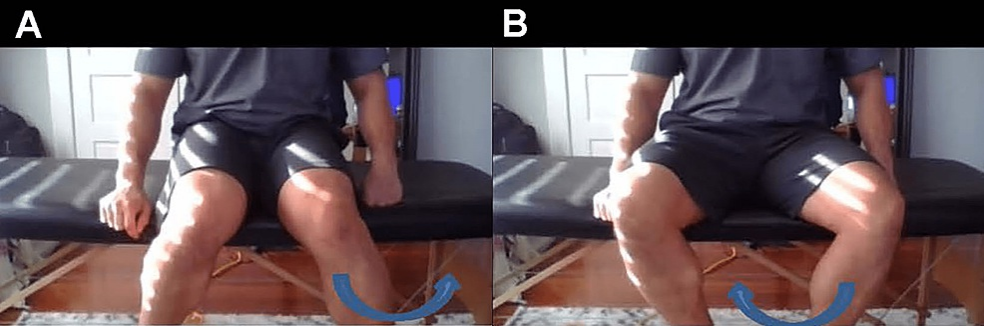
Hip rotation range of motion A. To assess internal rotation of the hip, instruct the patient to sit in a neutral position and turn their foot outwards (arrow). B. To assess the external rotation of the hip, instruct the patient to sit in a neutral position and turn their foot inwards (arrow). For both motions, it is helpful to compare both sides at the same time.

ROM testing is an important part of a thorough hip evaluation. It should compare both the affected and unaffected sides. ROM testing for hip complaints should also assess the ROM of the lumbar spine. First, instruct the patient to sit in a chair with hips flexed to 90°. Move ankles inwards and outwards, assessing external and internal ROM of the hip while monitoring for pain (Figure 2). In this same position, ask the patient to flex the affected hip past 90°. Does this cause pain? Is the patient able to cross their legs? If so, ask them to cross their legs and allow the knee of their top leg to fall out to the side. Does this position cause pain? With the patient standing comfortably, ask them to lift their leg out to the side (estimate abduction), then extend their leg behind them (estimate extension). Lumbar ROM can be assessed with the patient in a seated position. Instruct the patient to bend forward to touch their toes to determine flexion, then have them lean backward to assess extension. When the patient is performing these motions, view the patient from the side to approximate the total degree of motion. A posterior view should be used for the evaluation of side bending and rotation; this position enables more anatomical landmarks to be used in the ROM assessment.

Special testing

**Figure 3:**
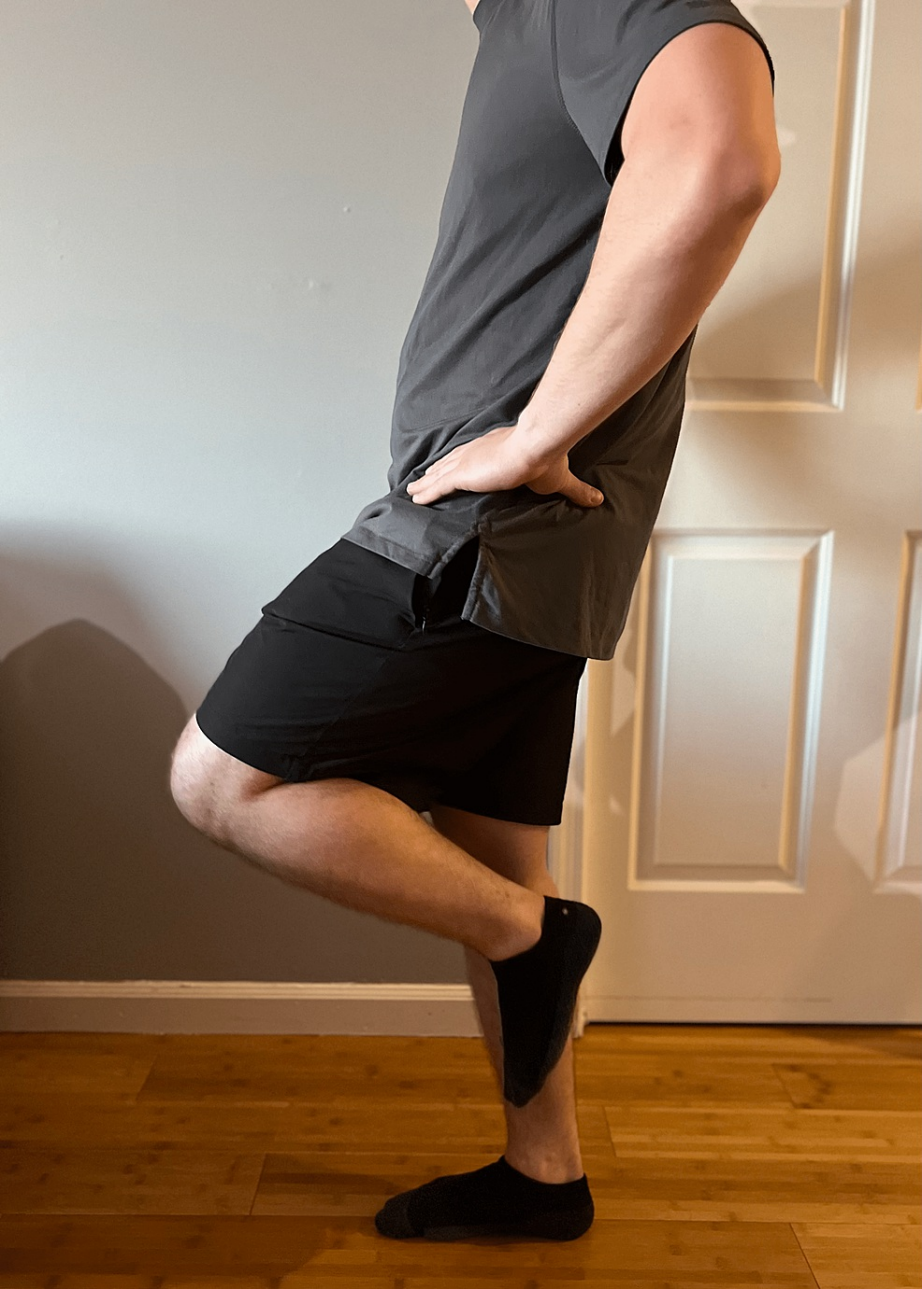
Stork test Instruct the patient to stand on one leg and hold the opposing heel to the stance leg. Pain in this position is a positive Stork test, suggesting pathology at the pars interarticularis, spondylolysis, or spondylolisthesis.

**Figure 4:**
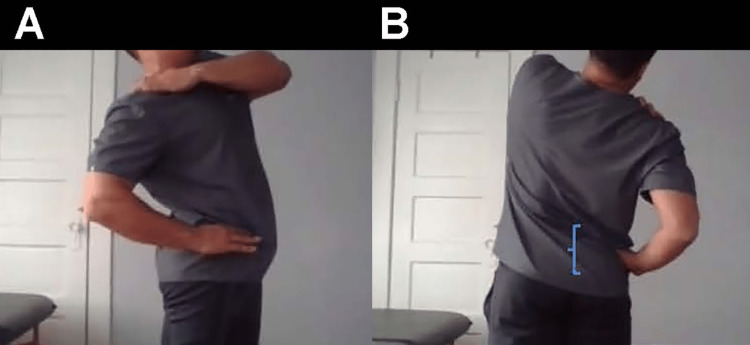
Modified lumbar extension loading (Kemp) test Instruct the patient to stand and bend to the affected side, using the contralateral hand to apply axial pressure to the spine. A. Lateral view, B. Posterior view. Lumbar facets (bracket) could be a somatic dysfunction or source of referred pain to the hip.

**Figure 5:**
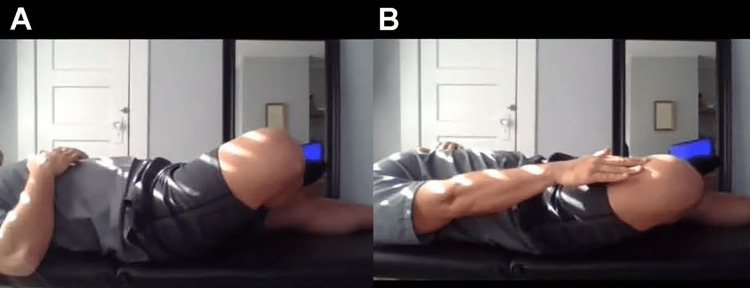
FABER test A. Instruct the patient to assume the figure-four position. Early pain suggests anterior hip joint pathology. B. Instruct the patient to apply downward pressure on the knee of the crossed leg pressure with their hand. Pain suggests sacroiliac or posterior hip pathology.

**Figure 6:**
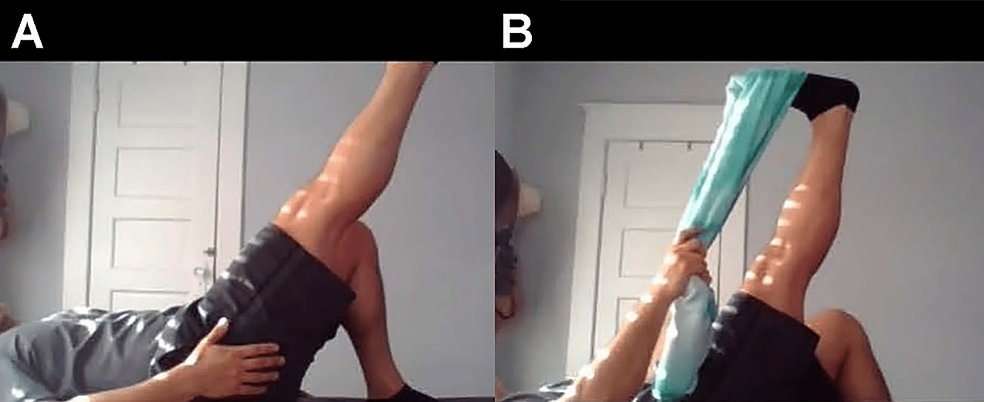
Modified straight leg and Bragard tests A. Instruct the patient to lie flat on their back and extend one leg, lifting the extended foot off the ground. If this produces pain, it is a positive straight leg raise test, suggesting lumbar pathology. B. Instruct the patient to maintain the position and flex the extended foot. If this produces pain, it is a positive Bragard test, suggesting lumbar radiculopathy or intra-articular pathology, depending on response.

**Figure 7:**
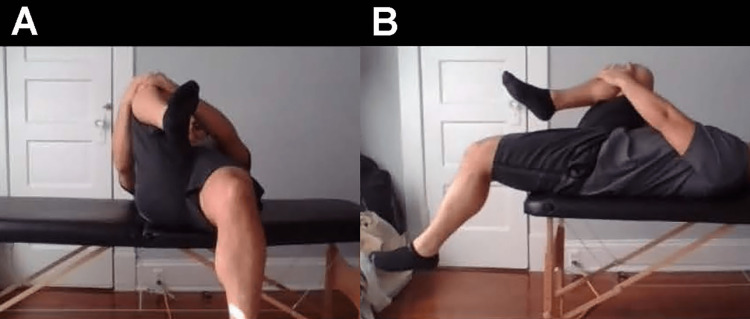
Thomas test A. Modified Thomas test from the anterior view. If the hip is flexed, there could be psoas involvement. If there is rotation, there may be iliotibial band restrictions or iliotibial band syndrome. If there is an abduction of the hip, there could be tensor fasciae latae involvement. B. Modified Thomas test from the lateral view. If the knee is extended, there could be rectus femoris involvement.

**Figure 8:**
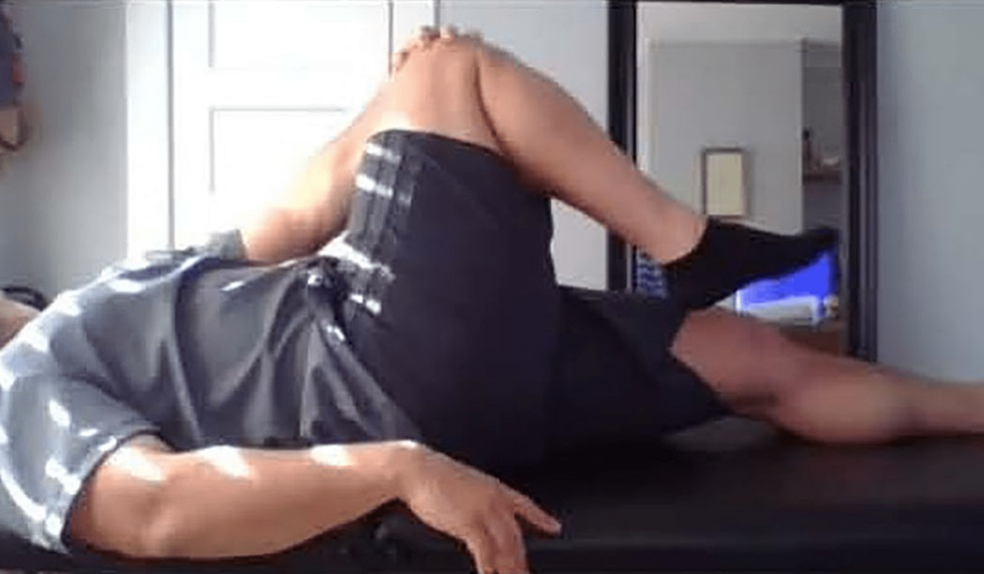
Modified FADDIR test Instruct the patient to flex, adduct, and passively move the hip from flexion to extension. Pain suggests osteoarthritis of the hip, femoral acetabular impingement, or anterior hip capsule pathology.

**Figure 9:**
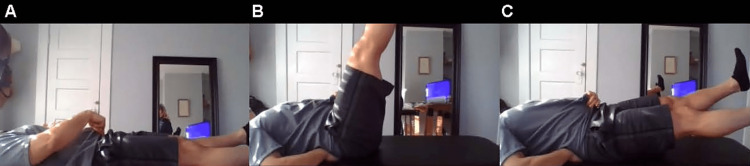
Pubic symphysis palpation and eccentric strength testing of the rectus abdominis A. Have the patient in a supine position palpate the pubic symphysis. Pain suggests athletic pubalgia. B. Instruct patient to raise their legs and place their hand nearest to the camera below the lumbar spine. C. Instruct the patient to slowly lower their legs, monitor for pain at the pubic symphysis, and note when the hand loses contact with the lumbar spine.

Special testing of the hip can be conducted in seated, standing, supine, and prone positions. Start with the patient in a seated position. From this position, the Beatty and Fortin finger signs can be determined. Next, have the patient transition to standing to complete the Stork and Kemp tests (Figures 3-4). Then, have the patient move to a supine position for the FABER, straight leg, Bragard, Thomas, and FADDIR tests (Figures 5-8). Also in this position, the palpation of the insertion of the rectus abdominus can be assessed to determine if there is pain (Figure 9). With the patient lying flat with legs extended, evaluate the outward rotation of the toes. Lastly, have the patient transition to a prone position to assess for gluteus medius tendinopathy, or dead butt syndrome, and assess the gluteal firing pattern. Having the patient perform multiple transitions from seated to standing to supine to prone and back to seated allows the physician to appreciate the overall impact the hip complaint has on the patient’s functional status.

## Results

Inspection and palpation

Guided questions and instructions for inspection and palpation are outlined in Table 1.

**Table 1:** Hip evaluation by telemedicine: inspection and palpation

What to say	Implications of an affirmative response
Inspection	
When standing facing the mirror with your hands on your hips, does one hip appear higher than the other? (Figure 1A)	Possible hip stabilizer weakness, anterior rotation of the hip, leg length discrepancy, hip flexor tightness, or sheared innominate
Looking at your hips in the mirror, does one hip appear to be more rotated than the other? (Figure 1B)	Possible anterior or posterior rotation of the hip or tightness in the hip flexor or hamstring
Do you see any differences between your left and right hip?	Possible fracture
Do you notice any a) sunken, b) swollen, c) bruised, or d) red areas on your hips?	Possible injury or infection; sunken areas could be areas of atrophy; swollen, bruised, or red areas may indicate injury ecchymosis
Palpation	
Lying on your side, palpate the lateral part of your hip. Does this cause pain?	Possible trochanteric bursitis or iliotibial band syndrome [9].
Palpate the area just below the dimple in your lower back/upper hip, does this cause pain? (Figure 1C)	Positive Fortin finger sign, suggesting sacroiliac joint pathology [10].
Do you have pain in your sits bones?	Possible ischial bursitis, hamstring avulsion, or hamstring tendinopathy [11].
Do you notice pain/numbness when you sit on your wallet? Are you able to reproduce this pain by pushing on the same area with your finger?	Possible piriformis syndrome [12].
Do you feel or see any groin masses?	Possible direct or indirect hernias [13].
Do you feel or see any mass in the front of your hip? (Figure 1D)	Possible femoral hernia [13, 14].
Do you feel pain when you press on the top of the pubic bone? (Figure 1D)	Possible athletic pubalgia [13].

Range of motion assessment and special testing

A guide for questions and instructions for ROM assessment and special testing are outlined in Table 2.

**Table 2:** Hip evaluation by telemedicine: ROM assessment and special testing FAI: Femoroacetabular impingement; ITB: Iliotibial band; ROM: Range of motion.

What to say	Implications of an affirmative response
ROM assessment	
Sit in a chair with your hips at 90°, and move your left/right ankle in and out. Does this cause pain in your groin? (Figure 2)	Hip ROM restriction, possible intra-articular pathology, such as arthritis, labral tear, or FAI [3].
Stand straight and slowly side bend to your left then to your right. Does this cause radicular pain in your hip or back?	Lumbar ROM restriction, possible intra-articular pathology, such as arthritis, stenosis, somatic dysfunction of the lumbar spine, or sacroiliac joint [3].
Stand straight and slowly extend backward arching your back. Does this cause pain in your hip?	Possible lumbar facet arthropathy, lumbar radiculopathy, or decreased ROM in the lumbar spine
Stand straight, slowly bend forward, and try to touch your toes. Does this cause radiating pain in your hip?	Possible lumbar disc pathology, lumbar radiculopathy [15].
Stand straight and slowly extend your hip. Does this produce pain or a snapping sensation in your hip?	Psoas, iliopsoas, rectus strain, and possible snapping hip syndrome [16].
Stand straight and slowly move your leg out to the side. Does this produce pain or tightness in your hip?	Possible adductor strain
Lie on your back and maximally flex your hip. Does this produce pain in your hip?	Decreased ROM in hip extensors or rotators, possible FAI [8].
Strength/special testing	
Lie flat on your back with both legs extended. Does one toe rotate out more than the other?	Possible hip stabilizer weakness, hypermobility, or somatic dysfunction of the innominate
Lie flat on your back with both legs extended. Slowly bring one foot up to the level of your knee and let your knee fall out to the side. Do you have any pain? (Figure 5)	Positive FABER test; if the pain is encountered early on, possible anterior hip joint pathology; if the pain is encountered later, possible posterior hip/sacroiliac joint pathology; if position elicits pain at the sacroiliac joint, possible sacroiliac dysfunction or sacroiliitis; if the pain is reproduced at the groin, possible iliopsoas strain, hip impingement, labral tear, chondral lesion, or osteoarthritis
Do you have any weakness, numbness, or shooting pain down your hip and leg?	Possible lumbar radiculopathy
Do you have numbness from your hip into the anterolateral aspect of your thigh?	Possible meralgia paresthetica [17]
In a seated position, put your affected leg over your unaffected leg’s knee (ie, cross your legs) and bend forward. Does this position reproduce your pain?	Positive modified Beatty test, suggesting piriformis syndrome [11,18]
Lie flat on your back with both legs straight in the air. Slowly lower your legs down. Do you notice pain? If so, where? Did your lower back come up off the ground? If so, when? (Figure 9)	Pain at the pubic symphysis, possible athletic pubalgia [13, 19].; early loss of contact of the lumbar spine from the ground during lowering of legs, possible weakness of the inferior aspect of the rectus abdominus or spinal stabilizers
Lie flat on your back, one leg extended. Lift one foot off the ground. Does this cause pain (Figure 6A)? Is it worsened by the dorsiflexion of your ankle? (Figure 6B)	Possible lumbar radiculopathy or intra-articular pathology, depending on response; positive Bragard test if increased radicular symptoms with ankle dorsiflexion [20]
Sit on the edge of the bed. Pull one knee towards your chest and lie flat on your back. Do you have pain in the hip that is straight? Do you notice a) hip flexion, b) knee extension, c) rotation of your lower leg, d) outward rotation of your hip? (Figure 7)	Positive modified Thomas test [21].; if the knee is extended, possible rectus femoris involvement; if the hip is flexed, possible psoas involvement; if rotation, possible ITB restrictions or ITB syndrome; if abduction of the hip, possible tensor fasciae latae involvement
Lie flat on your back, flex your hip, and passively move it up and down. Does this produce pain? (Figure 8)	Positive modified FADDIR test, suggesting FAI, osteoarthritis, or hip impingement [22].
Stand on one leg with your knee bent and slightly extend your spine. Does this produce pain? (Figure 3)	Positive Stork test, suggesting pathology at the pars interarticularis, spondylolysis, or spondylolisthesis [23].
Stand straight, extend your spine, then bend and rotate to the affected side. Does this produce pain or radicular symptoms? (Figure 6)	Positive lumbar extension loading test, suggesting pathology at the facet joints in the lumbar spine [24].
Stand and balance on your left/right leg. Slowly lower yourself into a single-leg squat. Does this cause pain? Do you feel unstable?	Possible hip stabilizer weakness, patellofemoral pain syndrome, or lumbar radiculopathy

Strength testing, functional assessment and gait analysis

**Figure 10:**
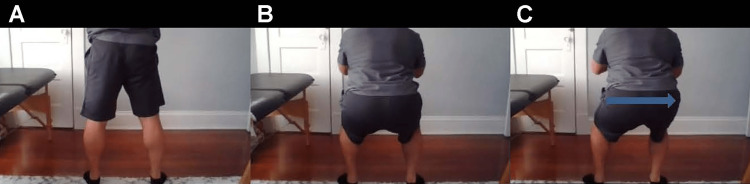
Hip shift A. Start position of the squat, posterior view. B. End position of squat with equal mobility of both sides. C. Positive hip shift to the right (arrow). The patient will shift to the affected side during the descent of the squat. The hip shift suggests weakness or reduced mobility in the affected hip.

Strength testing through a video visit is possible. It can be accomplished through active ROM and functional motions. For general lower body strength testing, have the patient perform a standard bodyweight squat. This motion should be assessed from both the lateral and posterior views. If the patient can perform this against gravity, their strength level is at least 3/5. Hip shifting tests the mobility and strength of the hip and should be assessed in a posterior view. If the patient shifts away from the affected hip, this is a positive hip shift test, indicating decreased mobility or strength in the affected hip (Figure 10). Next, have the patient stand on one leg and look for a positive Trendelenburg sign to test the strength of the gluteus medius, an important muscle in hip stabilization. This is indicated by the contralateral hip dropping and represents a weakness in the gluteus medius on the standing leg. Next, have the patient perform a single-leg squat, looking for hip weakness or dynamic genu valgus, and assess for pain.

**Figure 11:**
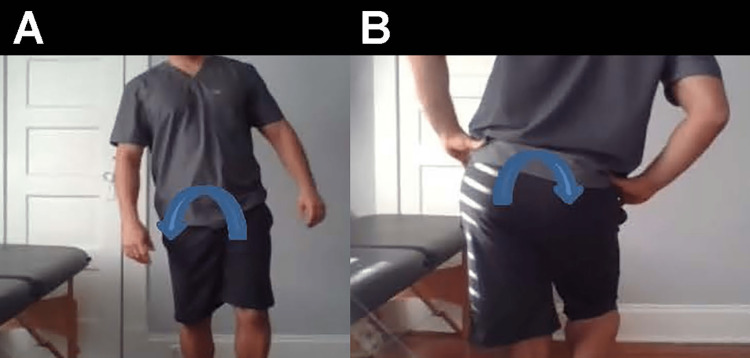
Trendelenburg gait Trendelenburg gait is caused by weakness in the stance leg hip abductor group and lack of eccentric control. The characteristic side bending is compensation to maintain pelvic stability in the gait cycle. A. Anterior view of gait side bending to patient’s right (arrow). B. Posterior view of gait side bending to patient’s right (arrow).

**Figure 12:**
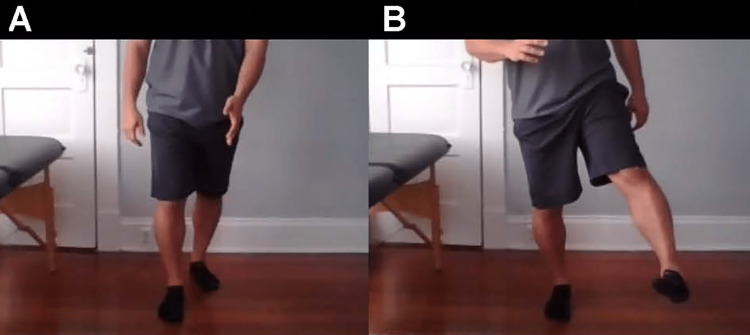
Circumduction gait A. Stance phase of gait. B. Swing phase of gait. Notice the exaggerated arc of the gait cycle, suggesting hypertonicity in the hip adductors.

**Figure 13:**
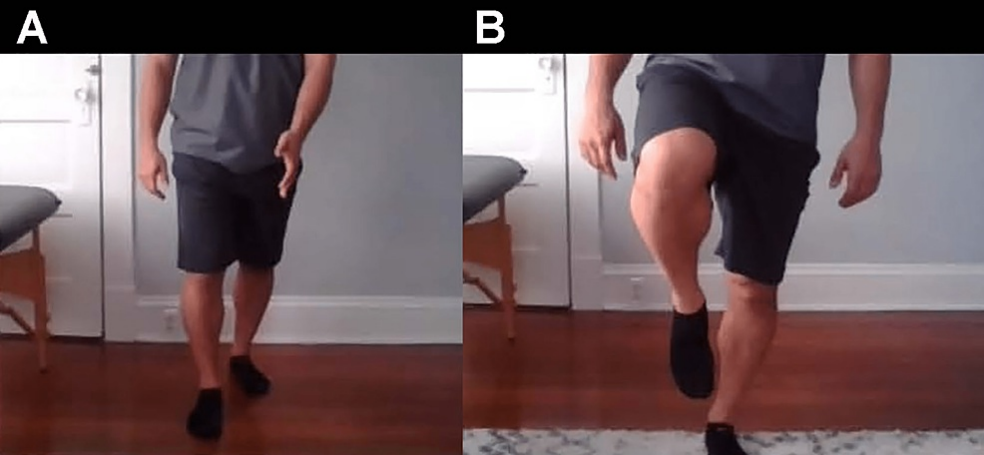
Steppage gait A. Stance phase of gait. B. Swing phase of gait, with increased hip flexion of the affected hip. Steppage gait pattern suggests nerve involvement. Possible diagnoses are spondylolisthesis, slipped femoral epiphysis, and disc herniation.

Functional assessment and gait analysis are valuable pieces of information to include in a thorough hip evaluation. Possible gait abnormalities that can be seen via video include Trendelenburg, circumduction, steppage, and antalgic gait patterns (Figures 11-13). During the gait examination, assess for stance width, stance time, and stride length. The gait pattern and functional assessment can be appreciated using the questions provided in Table 3.

**Table 3:** Hip evaluation by telemedicine: functional assessment, gait analysis, and special considerations

What to say	Implications of responses
Functional assessment and gait analysis	
Have you been limping?	Antalgic gait pattern, suggesting possible fracture muscle strain or joint pathology
Does your hip drop when you walk? (Figure 11)	Positive Trendelenburg sign, suggesting weakness in the gluteus medius of the affected limb [25].
Do you swing your leg out to the side when you walk? (Figure 12)	Circumduction gait pattern, suggesting weakness and spasticity in the hip and knee flexors or the dorsiflexors of the affected limb [26].
Do you flex your hip more than normal when you walk? (Figure 13)	Steppage gait, suggesting nerve involvement; possible spondylolisthesis, slipped femoral epiphysis, or disc herniation [27].
When you squat down, do you favor the unaffected side? (Figure 10)	Positive hip shift, suggesting weakness or reduced mobility in the affected hip [28].
Special considerations	
Does it hurt to lie on your left/right hip?	Possible trochanteric bursitis or iliotibial band syndrome
Do you have pain in your hips upon waking? Does pain gradually improve with movement?	Possible hip osteoarthritis

## Discussion

Indications for imaging and special considerations for telehealth

A low threshold for hip imaging should be considered in the following situations: inability to bear weight, severely antalgic gait, hip swelling or erythema, trauma to the hip, nighttime pain, and fever or chills with pain. Furthermore, loss of bladder or bowel control, loss of inner thigh sensation, and leg weakness warrant immediate in-person evaluation and imaging, likely magnetic resonance imaging or computed tomography.

Intra-abdominal conditions can cause referred pain in the anterior hip. To determine if this is the cause, a thorough history of the present illness and a review of historical medical problems and procedures can greatly aid the evaluation. Additionally, if the patient is female, evaluation of cystic ovaries, ovarian torsion, pelvic pain secondary to endometriosis, and myofascial pelvic pain should be considered in the differential diagnosis.

In order to utilize telehealth, patients and providers alike need access to a reliable Internet connection and access to internet-enabled devices with video capability. The digital infrastructure to support telehealth requires elevated security measures to protect patient health information at Health Insurance Portability and Accountabililty (HIPAA) standards. When using new technology, both the patient and provider need the skills and knowledge in order to communicate effectively. Provider education and training should be provided by the health system in which they work. Exam templates are helpful tools that can be built into provider workflows to improve their ability to perform telehealth examinations.

Limitations

While telehealth examination techniques are rapidly evolving and becoming more standardized, there are still limitations when compared to physical examination. A provider would not be able to use tools such as palpation or strength testing in their evaluation. Additionally, other conditions, such as those mentioned above, would not be able to be thoroughly evaluated via telehealth. In the case of a potentially serious injury, telehealth may delay care for the patient if it is decided a physical examination in person is needed. There is also the potential for user error; while procedures can be standardized and refined as much as possible, there remains the possibility that the patient is not able to self-perform a physical exam maneuver as accurately as a trained provider. Lastly, some limitations exist in the generalizability of this exam to all populations. Some of the outlined exam maneuvers may be difficult for certain patients with extremely limited mobility and may need to be further modified on an individual basis. Additionally, the telehealth exam relies heavily on clear and effective communication by the provider to ensure the patient is able to perform exam maneuvers correctly. As with in-person visits, but potentially magnified during telehealth visits, language barriers may lead to suboptimal participation in the outlined telehealth examination, and therefore, utilization of medical translation services is essential in these cases.

## Conclusions

Musculoskeletal considerations are important for patients with hip pain. The ability to evaluate hip complaints through telemedicine can be helpful for patients and providers alike. Patient satisfaction will continue to play an important role in patients becoming more comfortable with participating in telemedicine encounters in the future. Evaluation, assessment, and treatment of hip pain using telemedicine are beneficial when the patient is unable to drive or local access to a clinician is limited. Telemedicine evaluations are a cost and time-effective tool to improve patient access for the evaluation and management of common hip complaints.
